# Application of multiple occupational health risk assessment models in the prediction of occupational health risks of n-Hexane in the air-conditioned closed workshop

**DOI:** 10.3389/fpubh.2022.1017718

**Published:** 2022-12-07

**Authors:** Jiawei Zhu, Shibiao Su, Cuiju Wen, Tianjian Wang, Haijuan Xu, Ming Liu

**Affiliations:** Guangdong Province Hospital for Occupational Disease Prevention and Treatment, Guangzhou, Guangdong, China

**Keywords:** occupational health, occupational poisoning, risk assessment, n-Hexane, air-conditioned workshop

## Abstract

**Background:**

n-Hexane (NH) poisoning is a common occupational poisoning in the hardware and electronics industries. However, there is few research data on risk assessment of positions using NH in enclosed workshops. It is very important to assess the risk level of these positions and put forward effective measures and suggestions.

**Methods:**

The information of selected companies and air samples were collected through on-site investigation, and data collation and sample testing were carried out according to the requirements of Chinese standards. The Control of Substances Hazardous to Health (COSHH) Essential, the EPA non-carcinogenic risk assessment model, the Singapore exposure index method and the Chinese semi-quantitative risk assessment models were used to assess the risks of NH.

**Results:**

The working hours of the exposure groups, printing groups and packing groups all exceeded 9 h per day, less than 30% of each similar exposure groups (SEG) was equipped with the local exhaust ventilation, and 11.1% of the cleaning group and 8.3% of the printing group had NH concentrations in the air that exceeded the Chinese occupational exposure limit (OEL). In the EPA non-carcinogenic risk assessment model, each SEG was evaluated at high risk. In the Chinese semi-quantitative risk assessment models, all of the work groups of exposure groups, 91.7% of the work groups of printing groups, 77.8% of the work groups of printing groups, and 57.1% of the work groups of printing groups were evaluated at unacceptable risk. More than 40.0% of the work groups of printing groups and cleaning groups and over 20.0% of the work groups of exposure groups and packing groups were evaluated at high risk in the Chinese semi-quantitative risk assessment models.

**Conclusions:**

The Chinese exposure index method and the synthesis index method may have a stronger practicability. Some work groups that use NH in air-conditioned enclosed workshops in China, especially the cleaning groups, are still in a high-risk state. It is necessary to increase protective measures and strengthen occupational hygiene management to reduce risks.

## Background

NH is a colorless organic compound that belongs to the straight-chain saturated hydrocarbon (1). It is considered a low-boiling chemical that is volatile at room temperature because of its boiling point of 69°C and vapor pressure of 127.5 mmHg at 25°C. It has the advantages of low price and good performance, and thus it is widely used in various production processes. For example, it is used as a cleaning agent and adhesive in printing, hardware and electronic equipment manufacturing industries (2, 3). Workers are exposed to NH at work through inhalation, ingestion and skin contact (4), which is mainly metabolized to 2,5-Hexanedione(2,5-HD) in the body. The concentration of 2,5-HD in urine is often used as a biological monitoring indicator for workers exposed to NH (5). The occupational NH poisoning is mainly chronic or sub-chronic, with clinical manifestations of Polyneuropathy, including bilaterally symmetrical sensory abnormalities, sensory loss and weakness in lower extremities, and neurogenic damage related to electrophysiological changes (6). However, there is no effective treatment for NH poisoning currently. The widespread use of NH in many countries has led to polyperipheral neuropathy, and its poisoning incidents have been reported in China, the United States, Japan, and Italy (6–8). Shenzhen City in Guangdong Province is one of the concentrations of electronic processing industries in China. These manufacturers are dominated by small workshops with high NH use, lack of protection and poor management. Therefore, many cases of NH occupational poisoning have been reported. According to statistics from 2006 to 2011, NH poisoning accounted for 28.2% of the total occupational diseases in Shenzhen (9).

Volatile chemical poisons can exist in the production environment in gaseous form. The chemical poisons in the air are inhaled into the human body through the respiratory tract, and their degree of harm to the human body is closely related to their concentration. Due to the closed structure and lack of natural ventilation, the supply of fresh air and the discharge of polluted air are limited in the closed workshop. This makes the volatile poisons in the workshop easy to accumulate, and high concentrations of poisons in the air can cause poisoning or even death of workers (10). In addition, the closed workshops prefer to be equipped with air conditioners. The air-conditioned air is discharged after the indoor air is circulated and cooled, which cannot remove toxic chemicals in the air. Therefore, the air circulated in the workshop can promote the process of chemical accumulation in the air and the closed air-conditioned workshops are more prone to occupational poisoning than other types of workshops.

The occurrence of NH occupational poisoning associated with various factors, including long exposure duration, enclosed working environment, poor ventilation or low efficiency of the air circulation system, and insufficient protective measures (4, 11). The Occupational Health Risk Assessment (OHRA) model has been proposed as a tool to predict and control the health risks of occupational hazards, which predicts the possibility and extent of hazards by qualitative, quantitative and semi-quantitative methods, and proposes corresponding preventive and control measures (12). Currently, the U.S. Environmental Protection Agency (EPA), Singaporean, Australian, Romanian, International Council on Mining and Metals (ICMM), and UK Control of Substances Hazardous to Health (COSHH) Essential models are considered as the six most common OHRA models (13). Previous studies have analyzed the advantages and limitations of each model (14). Quantitative, semi-quantitative and qualitative methods can be used in combination to assess risk levels more accurately. China has issued the occupational health standard, the “Guidelines for occupational health risk assessment of chemicals in the workplace (GBZ/T 298-2017)” (15), which introduced the basic definition, content and specifications for the use of OHRA. The qualitative and quantitative models in the standard are based on the same principles as the COSHH basic model and the EPA model, respectively. The semi-quantitative model in the standard includes three methods: exposure ratio method, exposure index method and synthesis index method. The principle of exposure ratio method is the same as that of the Singapore model, while the exposure index method and comprehensive index method are further developed based on the Singapore model (16).

Given the excellent oil solubility and low price of NH, there are still many small and medium-sized enterprises in China using NH as auxiliary production material, especially in the Pearl River Delta region where the electronics, hardware and printing industries concentrated. Moreover, NH is still used in large quantities in India, Vietnam and other manufacturing-oriented countries. To sum up, Occupational poisoning of NH is still an important occupational health problem. This study aimed to assess the occupational health risk of NH in the electronics, hardware and printing industries in China by multiple OHRA models, and then propose risk control and hazard management measures to reduce the risk of exposure.

## Materials and methods

### Description of the similar exposure groups

In this study, factors such as the composition of chemicals used, the amount of use, the setting of positions and the number of people in the positions were considered as the inclusion criteria for companies. A total of 36 positions in 28 companies in Shenzhen City of Guangdong Province in China were selected as the research object, mainly in the electronics and printing industries. In these industries, NH chemicals were used as decontamination detergents for cleaning. According to the characteristics of each position and the similarity of the job content, 36 positions were divided into 4 groups. The SEGs included 8 exposure groups, 12 printing groups, 10 cleaning groups, and 6 packing groups. The work of the exposure groups are to use film cleaner to remove stains on the film, which is mainly distributed in the electronic industry. The work of the printing groups are to use ink cleaning agents such as screen washing agent to remove ink, which is mainly distributed in the printing and electronic industries. The work of the cleaning groups are to use cleaning agents such as wiping water to remove surface stains. The work of the packing groups are mainly to use detergent to remove surface stains during packaging and checking. Details including duration of work, usage of NH, exposure duration, the automation level, ventilation, first-aid facilities, personal protective equipment, emergency rescue measures, occupational health management and NH concentration levels in each group were included in the investigation.

### Site survey and on-site testing

We collected data through on-site surveys, including the number of workers, working hours, daily use of NH, exposure time and protective equipment, and then recorded the above information in questionnaire. The collection of air samples and the testing of laboratory NH samples were performed according to the methods described in Chinese Standards “Specifications of air sampling for hazardous substances monitoring in the workplace (GBZ159-2004)” (17) and “Determination of alkanes in the air of workplace (GBZ/T 160.38-2007)” (18). The 8-h time-weighted average concentration (C-TWA) and short-term exposure concentration (C-STEL) of NH were tested and compared with the permissible concentration-time weighted average (PC-TWA) and permissible concentration-short term exposure limit (PC-STEL) in the Chinese standard, “Occupational exposure limits for hazardous agents in the workplace Part 1: Chemical hazardous agents (GBZ 2.1-2019)” (19).

### Occupational health risk assessment models

The COSHH essential model, EPA non-carcinogenic risk assessment model, Singapore exposure index method and semi-quantitative risk assessment model in China were selected to assess the occupational health risk of NH. The semi-quantitative risk assessment model can be referred to the standard in China, “Guidelines for occupational health risk assessment of chemicals in the workplace (GBZ/T298-2017)” (15).

(1) The COSHH Essential model. This model conducts risk assessment through both health hazards and exposure levels of chemicals. Health hazards were determined by the range of occupational exposure limits (OELs) or by assigning the assessed substance to a hazard band using a Risk-phrase. The exposure level was determined by the physical property, such as volatility, and by the use of substance.
(2) The EPA non-carcinogenic risk assessment model. The non-carcinogenic risk level could be calculated by the following equation: *HQ* = *EC*/*RfC* (HQ = the hazard quotient, which is the value of the non-carcinogenic risk; EC = the exposure concentration for the acute exposure period; RfC = the reference concentration for inhalation toxicity).

In the equation, the RfC value of NH was 2 × 10^−3^ mg/m^3^. EC values were estimated based on the concentration of chemicals in the air, exposure duration and frequency, working age and etc. The HQ value was used to determine the risk level. When the value was greater than or equal to 1, it indicated that the chemical substance might have a high non-carcinogenic risk (unacceptable risk). In addition, when the value is < 1, it indicated that the chemical substance might have a low non-carcinogenic risk (acceptable risk).

(3) The Singapore exposure index method. This method is one of the methods of the Singapore model. The risk level can be calculated by the equation: $Risk\;=\;\sqrt{HR\times ER}$ (HR = hazard rating; ER = exposure rating).

In the equation, the HR values could be determined by the carcinogenicity classifications established by the American Conference of Government Industrial Hygienists (ACGIH) and the International Agency for Research on Cancer (IARC) or by the median lethal dose (LD50) and the median lethal concentration (LC50) of chemical substances in Material Safety Data Sheets (MSDS). The ER was calculated using equation: $ER\;{\left[EI_{1}\times EI_{2}\times \cdot \cdot \cdot EI_{n}\right]}^{1/n}$ (EI = the exposure index; n = the number of exposure factors, which includes vapor pressure, particle size, hazard control measures, weekly usage of the chemicals, and duration of work per week).

(4) The semi-quantitative risk assessment model in China. The model includes the exposure ratio method, exposure index method and synthesis index method. The risk level was calculated in the same way as the Singapore exposure index method.

In the exposure ratio method, the ER was determined by the ratio of the exposure level (E) and OEL, and the E was calculated using the equation: *E* = *F*×*D*×*M*/*W* (F = the frequency of exposure per week; M = the magnitude of exposure; W = the average working hours per week; D = the average duration of each exposure). Compared with the Singapore exposure index method, the EI of the Chinese exposure index method takes into account more factors, including first aid facilities, PPE, emergency rescue measures, occupational health management, daily use of chemicals and daily working hours. And in the synthesis index method, the ratio of exposure level to OEL (E/OEL) was added to the EI.

### Risk ratio conversion

The risk level was converted into the risk ratio with the equation: RR = R/N (RR = the risk ratio; R = the risk level; N = the number of total levels). In this study, the hazard ratios were divided into 5 ranges and defined as 5 adjusted risk levels (0–−0.2 = level 1; 0.2–0.4 = level 2; 0.4–0.6 = level 3; 0.6–0.8 = level 4; 0.8–1 = level 5).

### Statistical analysis

SPSS 22.0 software (IBM, Armonk, NY, USA) was used for statistical analysis. The statistical significance of differences between the groups was determined by one-way analysis of variance (ANOVA), followed by the Tukey *post hoc* test. The consistency of the two occupational health risk assessment models was assessed by Cohen's Kappa (k ≥ 0.75, indicating good consistency; 0.75 > k ≥ 0.40, indicating average consistency; k < 0.40, indicating lack of consistency).

## Results

### On-site survey and test results

The number of workers per group, working hours, usage of NH, automation level, ventilation measures, first-aid facilities, emergency rescue measures, occupational health management, and NH concentration levels for the SEGs were listed in Table 1. According to the results of the on-site investigation, the production processes were mainly semi-automatic and manual operations. Most of workplaces were set up with comprehensive ventilation system, while the rest of small number of manufacturers were equipped with local ventilation facilities for operating positions only. Most of manufacturers provided personal protective equipment for their employees, however, it was found that some of the PPE were not equipped in accordance with the standard requirements. In addition, most manufacturers had first-aid facilities and emergency rescue measures. The processes in the exposure and printing groups were mainly semi-automatic operations, while those in the cleaning and packaging groups were mainly manual operations. The amount of hexane used in the exposure and printing groups was significantly more than that in the cleaning and packaging groups. The C-TWA for hexane ranged from 4.20 to 70.30 mg/m^3^ with an average value of 20.76 mg/m^3^ in the exposure group, from 0.50 to 160.44 mg/m^3^ with an average value of 31.07 mg/m^3^ in the printing group, 0.40 to 100.40 mg/m^3^ with an average value of 41.29 mg/m^3^ in the cleaning group, and 1.60 to 33.20 mg/m^3^ with an average value of 13.79 mg/m^3^ in the packing group, The C-STEL of NH for hexane ranged from 4.90 to 82.90 mg/m^3^ with an average value of 34.51 mg/m^3^ in the exposure group, from 3.08 to 630.80 mg/m^3^ with an average value of 82.93 mg/m^3^ in the printing group, 0.40 to 265.30 mg/m^3^ with an average value of 86.76 mg/m^3^ in the cleaning group, and 5.60 to 89.40 mg/m^3^ with an average value of 54.61 mg/m^3^ in the packing group.

**Table 1:** Survey results of SEGs exposed to n-Hexane.

**SEG**		**Exposure groups**	**Printing groups**	**Cleaning groups**	**Packing groups**
Number of groups		7	12	9	7
Number of workers per group		4–53	2–35	1–11	2–8
Duration of work (months)		44.1(17–68)	32.8(6–156)	19.6(5–42)	20.7(6–48)
Daily usage (kg/L)		7.4 (0.2–20)	8.4 (0.1–30)	1.5 (0.25–4)	3.2 (0.005–15)
Weekly usage (kg/L)		44.1 (1.2–120)	48.1 (0.5–180)	8.6 (1.25–24)	18.9 (0.03–90)
Hours of work per day		9.7 (8–10)	9.3 (8–10)	7.0 (2–10)	9.3 (8–10)
Days of work per week		5.7 (5–6)	5.8 (5–6)	5.4 (5–6)	5.9(5–6)
C-TWA (mg/m^3^)		20.76 (4.20–70.30)	31.07 (0.50–160.44)	41.29 (0.40–100.40)	13.79 (1.60–33.20)
C-STEL (mg/m^3^)		34.51 (4.90–82.90)	82.93 (3.08–630.80)	86.76 (0.40–265.30)	54.61 (5.60–89.40)
E/OEL		0.557 (0.042–1.873)	1.173 (0.067–9.337)	0.935 (0.002–3.925)	0.631 (0.245–1.323)
**Result**					
	C-TWA disqualified	0 (0/7)	8.3% (1/12)	11.1% (1/9)	0 (0/7)
	C-STEL disqualified	0 (0/7)	8.3% (1/12)	11.1% (1/9)	0 (0/7)
**Automation level**					
	Full automation	0 (0/7)	0 (0/12)	0 (0/10)	0 (0/7)
	Semi-automation	100.0% (7/7)	66.7% (8/12)	33.3% (3/9)	14.3% (1/7)
	Manual operation	0 (0/7)	33.3% (4/12)	66.7% (6/9)	85.7% (6/7)
**Ventilation**					
	General ventilation	71.4% (5/7)	83.3% (10/12)	77.8% (7/9)	85.7% (6/7)
	Local exhaust ventilation	28.6% (2/7)	16.7% (2/12)	22.2% (2/9)	14.3% (1/7)
**First-aid facility equipped**		100% (7/7)	100% (12/12)	100% (9/9)	100% (7/7)
**Personal protective equipment**					
	Equipped	85.7% (6/7)	83.3% (10/12)	88.9% (8/9)	85.7% (6/7)
	Used or worn	85.7% (6/7)	83.3% (10/12)	88.9% (8/9)	85.7% (6/7)
**Emergency rescue measures complete**		85.7% (6/7)	58.3% (7/12)	77.8% (7/9)	85.7% (6/7)
**Occupational health management**				
	Performs well	71.4% (5/7)	75.0% (9/12)	77.8% (7/9)	71.4% (5/7)
	Performs poorly	14.3% (1/7)	25.0% (3/12)	22.2% (2/9)	28.6% (2/7)
	Lack of management	14.3% (1/7)	0 (0/12)	0 (0/9)	0 (0/7)

C-STEL, short-term exposure concentration; C-TWA, 8-h time weighted average concentration; E/OEL, the ratio of exposure concentration to the occupational exposure limit; the results here represent the larger ratios of C-TWA/PC-TWA and C-STEL/PC-STEL. PC-TWA, the permissible concentration-time weighted average; PC-STEL, the permissible concentration-short term exposure limit; SEG, the similar exposure group. ^*^P < 0.05 compared to degreasing groups.

Although the average values of C-TWA and C-STEL were higher in the printing groups and cleaning groups, the numerical differences between the SEGs were not statistically significant. In addition, the results of the survey showed that 8.3% of the printing groups and 11.1% of the cleaning groups had results of C-TWA and C-STEL exceeding the PC-TWA and PC-STEL in the Chinese standard.

### Risk assessment results

As shown in Table 2, NH had a risk level of R48, which indicated a risk of serious damage to health through long-term exposure through inhalation, dermal absorption and ingestion, and therefore its hazard class (HR) in the COSHH Essential model was considered to be Level D. Based on the volatility and use of NH in different manufacturers, the exposure rating (ER) of each SEG was grade 2 to 3. Combining the results of HR and ER, the COSHH essential model showed that all groups exposed to NH had a very high risk. Besides, the results of the EPA non-carcinogenic risk assessment model showed that a total of 82.9% of the all work groups had HQs >1, with 100% of the exposure groups, 91.7% of the printing groups, 77.8% of the cleaning groups and 57.1% of the packaging groups were >1. These results indicated that most work groups had high non-carcinogenic risks.

**Table 2:** Evaluation results of the COSHH Essential model and the EPA non-carcinogenic risk assessment model of n-Hexane.

**SEG**	**Number of groups**	**COSHH essential model**			**EPA non-carcinogenic risk assessment model**		
		**HR**	**ER**	**Risk level**	**HQ**	**Unacceptable risk ratio**	**Acceptable risk ratio**
Exposure groups	7	D	2–3	4 (Very high risk)	10.42 (1.93–32.10)	100.0% (7/7)	0
Printing groups	12	D	2–3	4 (Very high risk)	13.51 (0.23–78.49)	91.7% (11/12)	8.3% (1/12)
Cleaning groups	9	D	2–3	4 (Very high risk)	12.14 (0.06–35.10)	77.8% (7/9)	22.2% (2/9)
Packing groups	7	D	2–3	4 (Very high risk)	5.90 (0.10–11.07)	57.1% (4/7)	42.9% (3/7)
Total	35	D	2–3	4 (Very high risk)	10.84 (0.06–78.49)	82.9% (29/35)	17.1% (6/35)

COSHH, UK Control of Substances Hazardous to Health; EPA, U.S. Environmental Protection Agency; ER, exposure rating; HR, hazard rating; HQ, the hazard quotient; SEG, the similar exposure group.

The results of semi-quantitative risk assessment models of NH were listed in Table 3. Four models were used to assess risk levels. The results of the exposure ratio method showed that the risk levels of all work groups were distributed in levels 2–4 with 17.2% of the work groups being at high risk (level 4), which included 14.3% in the exposure group, 16.7% in the printing group, 22.2% in the cleaning group, and 14.3% in the packaging group. In addition, most of the work groups were at medium risk (level 3), with 71.4% in the exposure group, 66.6% in the printing group, 66.7% in the cleaning group, and 85.7% in the packaging group. The results of the Singapore exposure index method showed that the risk levels of all work groups were distributed in levels 3–4 with 22.9% of the work groups being at high risk, which included 50.0% in the printing group, 11.1% in the cleaning group, and 14.3% in the packaging group. There was no exposure group at high risk. The results of the Chinese exposure index method showed that the risk levels of all work groups were distributed in levels 3–4 with 40.0% of the work groups being at high risk, which included 28.6% in the exposure group, 41.7% in the printing group, 55.6% in the cleaning group, and 28.6% in the packaging group. Meanwhile, 60% in the work groups were at medium risk, including 71.4% in the exposure groups, 58.3% in the printing groups, 44.4% in the cleaning groups and 71.4% in the packing groups. The results of the composite index method showed that the risk levels of the work groups were distributed in levels 3–4, and it differed from the results of the Chinese exposure index method only in the distribution of risk levels in the clean group. In the Synthesis index method, 44.4% of the clean group was at high risk and 55.6% was at medium risk.

**Table 3:** Evaluation results of semi-quantitative risk assessment models of n-Hexane.

**SEG**	**Number of groups**	**R**	**Exposure ratio method**	**Singapore exposure index method**	**Chinese exposure index method**	**Synthesis index method**
Exposure groups	7	2	14.3% (1/7)	0	0	0
		3	71.4% (5/7)	100.0% (7/7)	71.4% (5/7)	71.4% (5/7)
		4	14.3% (1/7)	0	28.6% (2/7)	28.6% (2/7)
Printing groups	12	2	16.7% (2/12)	0	0	0
		3	66.6% (8/12)	50% (6/12)	58.3% (7/12)	58.3% (7/12)
		4	16.7% (2/12)	50% (6/12)	41.7% (5/12)	41.7% (5/12)
Cleaning groups	9	2	11.1% (1/9)	0	0	0
		3	66.7% (6/9)	88.9% (8/9)	44.4% (4/9)	55.6% (5/9)
		4	22.2% (2/9)	11.1% (1/9)	55.6% (5/9)	44.4% (4/9)
Packing groups	7	3	85.7% (6/7)	85.7% (6/7)	71.4% (5/7)	71.4% (5/7)
		4	14.3% (1/7)	14.3% (1/7)	28.6% (2/7)	28.6% (2/7)
Total	35	2	11.4% (4/35)	0	0	0
		3	71.4% (25/35)	77.1% (27/35)	60% (21/35)	62.9% (22/35)
		4	17.2% (6/35)	22.9% (8/35)	40% (14/35)	37.1% (13/35)

R, risk level; SEG, the similar exposure group.

The Cohen's Kappa is generally used to evaluate the consistency of bidirectional ordinal classification data. However, the COSHH Essential model, the EPA non-carcinogenic risk assessment model and the semi-quantitative model had different classifications of risk levels. In order to make the models comparable, the risk levels of each model were converted into the RR, and further their adjusted risk levels were obtained. Their adjusted risk levels were listed in Table 4. As shown in Table 5, the consistency of risk assessment models was analyzed by the Cohen's Kappa. There was general consistency between the Exposure ratio method and the Singapore exposure index method, the Singapore Exposure Index method and the Chinese Exposure Index method, and the Singapore Exposure Index method and the Synthesis Index method. The Chinese exposure index method had good consistency with the Synthesis index method. The remaining Cohen's Kappa results suggested a lack of consistency.

**Table 4:** Risk ratio transformation for risk levels of multiple risk assessment models.

**SEG**	**Number of groups**	**RR**	**R (adjusted)**	**COSHH essential model**	**EPA non-carcinogenic risk assessment model**	**Exposure ratio method**	**Singapore exposure index method**	**Chinese exposure index method**	**Synthesis index method**
Exposure groups	7	0.2–0.4	2	0	0	14.3% (1/7)	0	0	0
		0.4–0.6	3	0	0	71.4% (5/7)	100.0% (7/7)	71.4% (5/7)	71.4% (5/7)
		0.6–0.8	4	42.9% (3/7)	28.6% (2/7)	14.3% (1/7)	0	28.6% (2/7)	28.6% (2/7)
		0.8–1	5	57.1% (4/7)	71.4% (5/7)	0	0	0	0
Printing groups	12	0.2–0.4	2	0	8.3% (1/12)	16.7% (2/12)	0	0	0
		0.4–0.6	3	0	0	66.6% (8/12)	50% (6/12)	58.3% (7/12)	58.3% (7/12)
		0.6–0.8	4	83.3% (10/12)	41.7% (5/12)	16.7% (2/12)	50% (6/12)	41.7% (5/12)	41.7% (5/12)
		0.8–1	5	16.7% (2/12)	50% (6/12)	0	0	0	0
Cleaning groups	9	0–0.2	1	0	11.1% (1/9)	0	0	0	0
		0.2–0.4	2	0	11.1% (1/9)	11.1% (1/9)	0	0	0
		0.4–0.6	3	0	0	66.7% (6/9)	88.9% (8/9)	44.4% (4/9)	55.6% (5/9)
		0.6–0.8	4	88.9% (8/9)	0	22.2% (2/9)	11.1% (1/9)	55.6% (5/9)	44.4% (4/9)
		0.8–1	5	11.1% (1/9)	77.8% (7/9)	0	0	0	0
Packing groups	7	0.2–0.4	2	0	14.3% (1/7)	0	0	0	0
		0.4–0.6	3	0	28.6% (2/7)	85.7% (6/7)	85.7% (6/7)	71.4% (5/7)	71.4% (5/7)
		0.6–0.8	4	85.7% (6/7)	14.3% (1/7)	14.3% (1/7)	14.3% (1/7)	28.6% (2/7)	28.6% (2/7)
		0.8–1	5	14.3% (1/7)	42.9% (3/7)	0	0	0	0
Total	35	0–0.2	1	0	2.9% (1/35)	0	0	0	0
		0.2–0.4	2	0	8.6% (3/35)	11.4% (4/35)	0	0	0
		0.4–0.6	3	0	5.7% (2/35)	71.4% (25/35)	77.1% (27/35)	60% (21/35)	62.9% (22/35)
		0.6–0.8	4	77.1% (27/35)	22.9% (8/35)	17.2% (6/35)	22.9% (8/35)	40% (14/35)	37.1% (13/35)
		0.8–1	5	22.9% (8/35)	60% (21/35)	0	0	0	0

RR, risk ratio; R, risk level; COSHH, UK Control of Substances Hazardous to Health; EPA, U.S. Environmental Protection Agency; SEG, the similar exposure group.

**Table 5:** Cohen's Kappa results of risk assessment models of n-Hexane.

**Cohen's Kappa (A Vs. B)**	**Value**	**Approx. Sig**.
Exposure ratio method vs. Singapore exposure index method	0.582	0.000
Exposure ratio method vs. Chinese exposure index method	0.318	0.012
Exposure ratio method vs. Synthesis index method	0.355	0.006
Exposure ratio method vs. COSHH Essential model	0.012	0.692
Exposure ratio method vs. EPA non-carcinogenic risk assessment model	0.058	0.155
Singapore exposure index method vs. Chinese exposure index method	0.615	0.000
Singapore exposure index method vs. Synthesis index method	0.668	0.000
Singapore exposure index method vs. COSHH essential model	-0.006	0.869
Singapore exposure index method vs. EPA non-carcinogenic risk assessment model	-0.043	0.276
Chinese exposure index method vs. synthesis index method	0.940	0.000
Chinese exposure index method vs. COSHH Essential model	0.050	0.324
Chinese exposure index method vs. EPA non-carcinogenic risk assessment model	-0.078	0.102
Synthesis index method vs. COSHH essential model	0.039	0.418
Synthesis index method vs. EPA non-carcinogenic risk assessment model	-0.072	0.124
COSHH Essential model vs. EPA non-carcinogenic risk assessment model	0.001	0.989

## Discussion

As a major component of cleaning agent, NH has been widely used in the manufacturing industry. Because NH has a high lethal dose (LD50 = 25 g/kg, orally administered in rats), it is considered as a low toxic compound, therefore, manufacturers have paid little attention to its toxicity. However, given the low boiling point of NH, it is readily absorbed at normal temperatures. Under the condition of massive long-term use of NH and improper protection, occupational poisoning is likely to occur, posing a threat to workers' health. This study targeted NH-exposed industries, including electronics, printing and hardware industries. Through on-site investigation and the application of multiple risk assessment models, the risk levels of different job positions were determined to provide a basis for risk management.

The results of the field survey showed that although the mean values of C-TWA and C-STEL of NH were the highest in the cleaning group with 41.29 and 86.76 mg/m^3^, respectively, the average usage were very small. This may be due to the fact that 66.7% of the workers in the cleaning group may have been directly exposed to chemicals containing NH during manual handling, resulting in more severe exposure. In the packaging group, 85.7% of the production processes required manual operation, but the average values of C-TWA and C-STEL in this group were only 18.9 and 45.09 mg/m^3^, respectively. Therefore, it can be speculated that most of the products may have been cleaned before the packaging process, reducing the NH exposure of packaging workers. In addition, the average NH use was higher in the exposure group and printing group but with normal results of C-TWA and C-STEL for each group, which could be accounted for the higher automated process level and fully equipped ventilation facilities. Only 8.3% of the printing groups and 11.1% of the cleaning groups had exposure concentrations higher than the Chinese occupational exposure limit, which may relate to the characteristics of the production process, automation level and the effectiveness of ventilation facilities.

We evaluated the occupational health risk of NH using three methods, namely COSHH Essential model, EPA non-carcinogenic risk assessment model and semi-quantitative risk assessment model (i.e., Singapore model and three semi-quantitative risk assessment models in China.) The result of COSHH Essential model showed that all the SEGs were at very high risk. According to COSHH Essential model, Since HR of NH was D, when the volatility was considered moderate, the ER of work groups were grade 2–3 with the risk levels 3–4. We took the highest risk level value as the outcome (very high risk). COSHH Essential model is relatively simple and easy to understand, but the drawbacks are obvious too, such as overestimation of the results and influence of subjectivity on judgement of liquid volatility. The EPA non-carcinogenic model is a quantitative assessment model, which can comprehensively evaluate the non-carcinogenic and carcinogenic risks of chemicals, but NH is non-carcinogenic and we do not need to access its carcinogenic risk. Compared to the COSHH Essential model, the EPA models is more reliable because the EPA model assesses risk level by adopting highly-weighted parameters, which tend to show a higher risk level (20). In the EPA's non-carcinogenic risk assessment model, the risk level is determined by the EC and the RfC of the toxicant. The RfC represents the reference concentration of continuous inhalation that does not cause some health risk over a lifetime. Even if both the RfC of NH and its concentration in air are low (< 0.5 OEL), the risk level is still high. In addition, the COSHH Essential model may overestimate the levels of risk from NH exposure in work groups because this model highly depends on the physicochemical property and exposure level of the substance but ignores the factors such as automation level, ventilation settlement, emergent rescue measures, management and utilization rate. Therefore, focus on workers' exposure to NH in the above manufacturers may not be sufficient. Nevertheless, the EPA non-carcinogenic risk assessment model can only classify risk level as high and low, leading it unable to distinguish different risk levels well.

Nonetheless, the EPA non-carcinogenic risk assessment models can only classify risk levels as high and low, resulting in a crude and vague assessment. The results of the semi-quantitative risk assessment model showed that the risk levels were 2 to 4 for the exposure, printing and cleaning groups and 3 to 4 for the packaging group. In the exposure ratio method, exposure concentration was the only factor taken into account for the risk levels, without considering the effects of protective measures. The Chinese exposure index method is used only in the absence of air monitoring data and is similar to the Singapore exposure index method, which focuses on exposure factors other than exposure concentrations. The Singapore exposure index method considers vapor pressure or particle size, hazard control measures, weekly chemical usage, and weekly working hours, while the Chinese exposure index method considers more specific exposure factors, including first aid facilities, personal protective equipment, emergency rescue measures, occupational health management, daily usage of chemicals, and daily working hours. The composite index method has an additional exposure concentration of another exposure factor based on the Chinese exposure index method. As shown in Table 3, in the cleaning groups, the evaluation results of the Singapore exposure index method for certain groups were lower than those of the Chinese exposure index method and the synthesis index method. No change in the results of other groups. In this study, some work groups of each SEGs was considered in a high-risk state because of high exposure concentration or low level of automation or poor occupational health management. In general, the risk of the cleaning groups were the highest. Compared to other two methods, the Chinese exposure index method and the synthesis index method were considered to be more practical, except that their relatively rough index classification. In order to improve the reproducibility of the risk assessment methods used in this study and promote the application of the risk assessment methods, the overview of the application of risk assessment methods was listed in Table 6. In the follow-up study, the risk level of NH poisoning in each post will be more accurately evaluated in combination with the population health data.

**Table 6:** An overview of the application of risk assessment methods relevant to this study.

**Classification**	**Model**	**Parameter**	**Equation**	**Advantage**	**Disadvantage**
Qualitative model	COSHH essential model	health hazard; *exposure levels.	Matrix method	The usage and nature of chemicals are considered. Simple and easy to implement	Protection measures and management measures are not considered; The results may be overestimated; The results may be influenced by subjectivity on judgement of usage of chemicals.
Quantitative model	EPA non-carcinogenic risk assessment model	*EC = the exposure concentration for the acute exposure period; RfC = the reference concentration for inhalation toxicity	*HQ* = *EC*/*RfC* (HQ ≥ 1, unacceptable risk; HQ < 1, acceptable risk)	Quantitative data can be well used, including the exposure concentration; The non-carcinogenic and carcinogenic risks of chemicals can be adequately assessed.	Protection measures and management measures are not considered; The results may be overestimated; The risk levels cannot be differentiated in detail.
	EPA carcinogenic risk assessment model	IUR = the inhalation unit risk; *d = the exposure dose that equals the chemical concentration in the air; t_E_ = the exposure duration; t_L_ = the life expectancy	$IR\;=\;IUR\times d\times \frac{t_{E}}{t_{L}}$ (IR≥10^−4^, unacceptable risk; IR < 10^−4^, acceptable risk)		
Semi-quantitative models	Exposure ratio method	*F = the frequency of exposure per week; *M = the magnitude of exposure; *W = the average working hours per week; *D = the average duration of each exposure; OEL = occupational exposure limit HR = hazard rating; ER = exposure rating	*E* = *F*×*D*×*M*/*W* $ER\;=\;\frac{E}{OEL}$ $Risk\;=\;\sqrt{HR\times ER}$	The exposure concentration is considered.	Protection measures and management measures are not considered.
	Singapore exposure index method			Protection measures and management measures are considered.	The classification of the exposure index is relatively crude. The exposure concentration is not considered.
	Chinese exposure index method	*EI = the exposure index; n = the number of exposure factors; HR = hazard rating; ER = exposure rating	$ER{\left[EI_{1}\times EI_{2}\times \cdot \cdot \cdot EI_{n}\right]}^{1/n}$ $Risk\;=\;\sqrt{HR\times ER}$	Protection measures and management measures are considered.	The classification of the exposure index is relatively crude. The exposure concentration is not considered.
	Synthesis index method			Protection measures and management measures are considered.	The exposure index classification is relatively crude.

^*^indicates that the data is obtained by on-site investigation.

In a survey in 2016, NH was detected in the production raw and auxiliary materials of 46 of the 61 companies using organic solvents in Shenzhen. These 46 companies were mainly distributed in the electronic industry and the printing industry, while the work groups exposed to NH were mainly cleaning groups, printing groups and exposure groups, etc. They used chemicals containing n-Hexane in the production process, such as wiping water, detergent, etc. (21). The industry distribution and position distribution were consistent with the investigation of this study. This survey results showed that the qualification rate of cleaning groups was 77.7%, the printing groups was 80.5%, and the packing groups was 86.6%. Therefore, the cleaning group had the most failure points and workers were more prone to occupational poisoning without proper protection. Since 2000, cases of NH occupational poisoning have been reported in some regions of China. As shown in Table 7, work groups, case number, working hours, ventilation facilities, personal protective equipment and occupational health management of the reports have been listed (22–27). It was found that the manufacturers where NH poisoning occurred had some common features, such as most cases were cleaning, packaging and printing workers, with the highest incidence among cleaning workers. This is consistent with the risk assessment results of Chinese exposure index method and synthesis index method. The results of both methods indicated that the cleaning group had a relatively high level of risk, so they were theoretically the most likely to have the highest number of poisoning events. These cleaning workers worked in an enclosed space without proper ventilation or sufficient personal protective equipment, and used hexane-based detergents for cleaning or wiping for more than 8 h per day. In terms of management, most companies do not have an established occupational health management system in place, nor are they hiring full-time management personnel with occupational health-related knowledge. To sum up, these cases shared some common feathers, such as long working duration, poor ventilation in workplace, and ineffective protection, etc. It suggested that the above factors that closely related to the risk of NH poisoning could be the common problems in most manufacturers using NH and can be used as critical control points to propose risk management measures to reduce the risk level.

**Table 7:** Information on studies in China related to n-hexane occupational poisoning.

**References**	**Work group**	**Number of cases**	**Working hours**	**Ventilation**	**Personal protective equipment**	**Occupational health management**
Zhou et al. (22)	Cleaning groups	58/58 (100%)	/	Most without local exhaust ventilation	/	/
Hu et al. (23)	Cleaning groups	13/13 (100%)	11–12 h/d, more than 6 days per week	poor general ventilation, no local exhaust ventilation	Some wear gloves and masks	Lack of management
Mao et al. (24)	Cleaning groups	24/24 (100%)	8 h/d	central air conditioning, no local exhaust ventilation	No personal protective equipment	Lack of management
Xuan et al. (25)	Cleaning groups	23/23 (100%)	8–10 h/d	No ventilation	Finger sleeves	/
Li et al. (26)	Printing groups	5/39 (12.8%)	/	/	/	/
	Cleaning groups	32/39 (82.1%)	/	/	/	/
Zhang et al. (27)	Cleaning groups	49/62 (79%)	10 h/d, more than 6 days per week	No ventilation or no use	No personal protective equipment	Perform poorly
	Packing groups	13/62 (21%)	10 h/d, more than 6 days per week	No ventilation or no use	No personal protective equipment	Perform poorly

Combined with the results of this study and related research, the proposed risk management measures are mainly aimed at companies and workers. For manufacturers, the most effective measure is to replace NH with low or non-toxic chemicals, such as medical alcohol, isopropanol, n-heptane. If NH cannot be replaced according to the production process, effective control measures should be taken. Such as improving mechanization, automation, confinement and remote operation of the process, reducing the chance of direct contact of manual work, adjusting working hours to reduce workers' contact time, avoiding the use of NH in air-conditioned workshops as much as possible and setting up effective local exhaust facilities to reduce NH concentration in the workplace. In addition, enterprises should strictly implement occupational health management, regularly monitor NH concentration in the workplace, conduct occupational health checkups for employees at least once a year, and provide workers with effective personal protective equipment, such as respirators and protective gloves. It has been proved that the NH concentration in the workplace air can be greatly reduced after using NH-free chemicals and installing efficient local ventilation facilities (28). On the other hand, workers need to raise their awareness of self-protection. For instance, they should stand in the upwind of the airflow as closed to the exhaust hood as possible without affecting operations. In addition, workers should properly wear personal protective equipment and seek medical treatments if physical abnormalities appear.

## Conclusions

The OHRA model in the Chinese standard GBZ/T 298-2017 can be used to assess the occupational health risks of NH, while the Chinese exposure index method and the synthesis index method may be more practical. Some work groups that use NH in the air-conditioned enclosed workshops in China are still in a high risk, especially printing groups and cleaning groups. It is critical to take risk management measures to reduce the risks.

## Data availability statement

The original contributions presented in the study are included in the article/supplementary material, further inquiries can be directed to the corresponding author/s.

## Author contributions

SS, CW, and ML contributed to the data acquisition, analysis, interpretation, drafted, and critically reviewed the manuscript for intellectual content. HX, JZ, and TWcontributed to the data analysis. SS and JZ conceived and designed the study. SS is the guarantor of this work and, as such, had full access to all the data in the study and takes responsibility for the integrity of the data and the accuracy of the data analysis. All authors reviewed and approved the final version of the manuscript.
